# Hyperbranched polyethylenimine bearing cyclodextrin moieties showing temperature and pH controlled dye release

**DOI:** 10.3762/bjoc.7.130

**Published:** 2011-08-18

**Authors:** Indra Böhm, Susanne Katharina Kreth, Helmut Ritter

**Affiliations:** 1 Institut für Organische und Makromolekulare Chemie II, Heinrich-Heine-Universität Düsseldorf, Universitätsstraße 1, D-40225 Düsseldorf, Germany Fax: (+49) 211 8115840

**Keywords:** anthraquinone, cyclodextrin, drug delivery, polyethylenimine, release

## Abstract

The release of anthraquinone dyes from β-cyclodextrin modified, hyperbranched polyethylenimine (PEI-CD) was investigated. 5,8-Dichloro-1,4-dihydroxyanthraquinone (AQ-OH) was enclosed simply by ionic attraction between the phenolate groups of AQ-OH and the protonated amino groups of polyethylenimine (PEI). Additionally, the adamantyl moieties of 1,4-bis-*N*-adamantylaminoanthraquinone (AQ-Ada) were encapsulated by the cavity of CD modified PEI. Due to these different types of interaction, the dyes can be controllably released from the CD-cavity (AQ-Ada) by temperature variation or from salt encapsulation by pH variation (AQ-OH), as monitored by UV–vis spectroscopy.

## Introduction

The development of novel materials and their use as drug delivery systems has gained increased interest in the last few decades [1–3]. In this context, certain stimuli responsive behaviors are required [4–5]. Hence, the ability to release molecules in response to temperature or pH change is an important feature of new biopharmaceutical compounds [6–11].

Hyperbranched polyethylenimine (PEI) is a promising material for medical applications due to its high biocompatibility [12–13]. Active drugs bearing anionic groups can interact with the cationic hyperbranched structure of the polymeric matrix [14]. These components can be released by changing the pH value [15]. In this context we recently reported the synthesis of hyperbranched PEI bearing covalently attached β-cyclodextrin (CD) [16]. CD has been exploited to complex various hydrophobic guests such as proteins, dyes and anti tumor drugs [17–22]. Inclusion complexes with, e.g., adamantane derivates, are relatively stable due to their high binding constants [23–24]. These kinds of supramolecular complexes become less stable under heating [25]. For optical visualization of the attachment and release of suitable molecules, two anthraquinone dyes were chosen as model compounds in the present work [26].

## Results and Discussion

5,8-Dichloro-1,4-dihydroxyanthraquinone (**1**) and 1,4-bis-*N*-adamantylaminoanthraquinone (**2**) were used as model compounds for a drug delivery system in combination with hyperbranched cationic polyethylenimine bearing covalently attached β-CDs **3**.

**Scheme 1:**
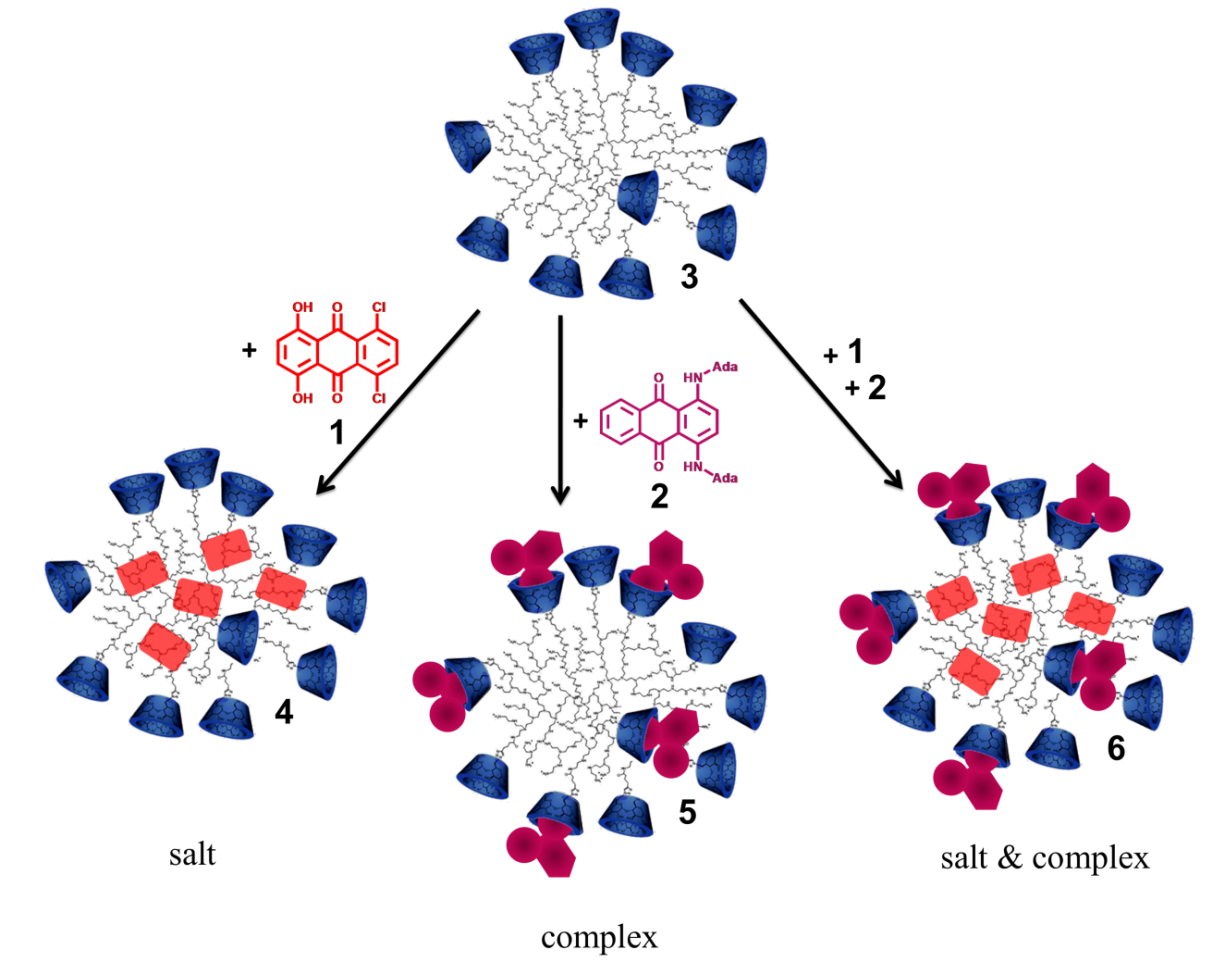
Preparation of salts and/or complexes **4**, **5** and **6**.

As outlined in Scheme 1, each dye, **1** and **2**, was mixed with **3** in water to form a salt **4** and a host–guest complex **5**, respectively. Additionally, both dyes were mixed together with **3** to provide complex **6** (Scheme 1).

The increase in hydrodynamic diameters (*d*_H_), measured by DLS, indicates the successful inclusion of both dyes **1** and **2** in polymer **3**. The inclusion of slightly acidic **1** in the cationic PEI induces an increase of *d*_H_ from 8 nm for compound **3** to 163 nm for the corresponding salt **4** (Supporting Information File 1, Figure S1) due to electrostatic expansion effects. In comparison, the *d*_H_ of unmodified PEI also increases by the inclusion of **1** (Supporting Information File 1, Figure S2). The combination of unmodified PEI and dye **2** could not be measured since the dye is not water-soluble and cannot be enclosed by PEI.

In contrast, the two hydrophobic adamantyl moieties of **2** were enclosed in the CD cavity of PEI, yielding complex **5**. An increased *d*_H_ from 8 nm to 260 nm due to intermolecular effects was measured by DLS (Supporting Information File 1, Figure S3). This effect strongly indicates the formation of higher aggregates due to intermolecular interactions. DLS measurements of the complex of unmodified, randomly methylated, β-cyclodextrin (RAMEB-CD) and dye **2** were performed, giving a *d*_H_ of 68 nm (Supporting Information File 1, Figure S6). The combination of RAMEB-CD and dye **1** could not be measured since the dye is not water-soluble and cannot be enclosed by CD.

Complex **6** exhibits enlarged networks with a *d*_H_ of 480 nm, which verifies an inclusion of both dyes (**1** and **2**) through salt formation and host-guest complexation (Supporting Information File 1, Figure S4).

**Figure 1:**
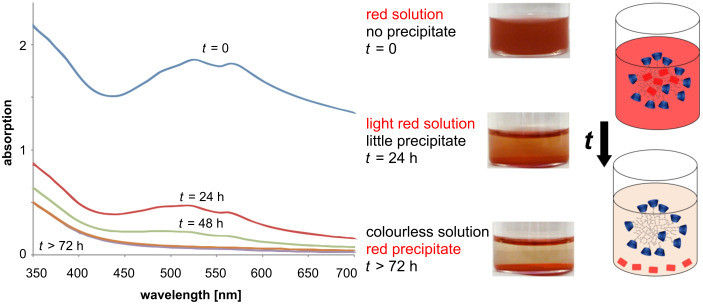
UV–vis spectra of complex **4** in basic solution (pH 10, *t* = 0 (blue line), *t* = 24 h (red line), *t* = 48 h (green line), *t* > 72 h (orange line and violet line)), and corresponding photos and schematic illustrations of the solution.

In water, a red solution of **4** was observed with λ_max_ at 524 and 564 nm. The water insoluble dye **1** becomes water soluble since the deprotonated hydroxy groups form a salt with the cationic amino moieties of PEI. The system reaches an equilibrium state between the complexed and the released dye **1** at pH values below 9. In basic solution no interaction takes place due to deprotonation of the amino moieties. Regarding the UV–vis spectra, the absorption intensity at λ_max_ (524 nm) for *t* = 0 corresponds to 100% of the dye being enclosed. Hence, 74% of the red dye was released after 24 h, a total amount of 88% after 48 h and a complete precipitate was observed after 72 h (Figure 1). This can be visualized even by the naked eye as the solution becomes light brownish (from the soluted polymer **3** which was also identified by DLS) and the red dye forms a residue.

The water insoluble dye **2** becomes water soluble as the hydrophobic adamantyl moieties are encapsulated by CD. That leads to a purple aqueous solution with λ_max_ at 552 and 668 nm. This complex **5** is not pH sensitive, which was verified by UV–vis spectroscopy (Supporting Information File 1, Figure S7).

**Figure 2:**
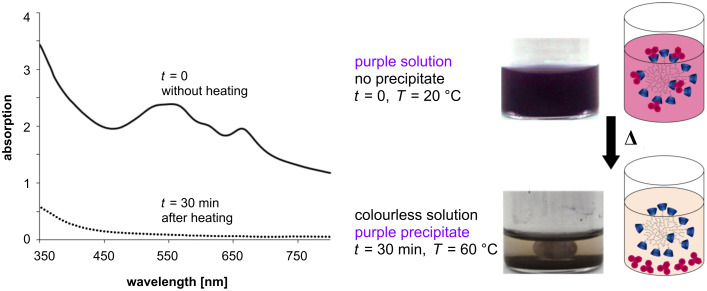
UV–vis spectra of complex **5** in solution before heating (solid line) and after 30 min of heating at 60 °C (dotted line), and corresponding photos and schematic illustrations of the solution.

After 30 min of heating at 60 °C the complexation equilibrium shifted towards the free purple colored dye **2**. The complete release was monitored by eye and by UV–vis spectroscopy (Figure 2).

**Figure 3:**
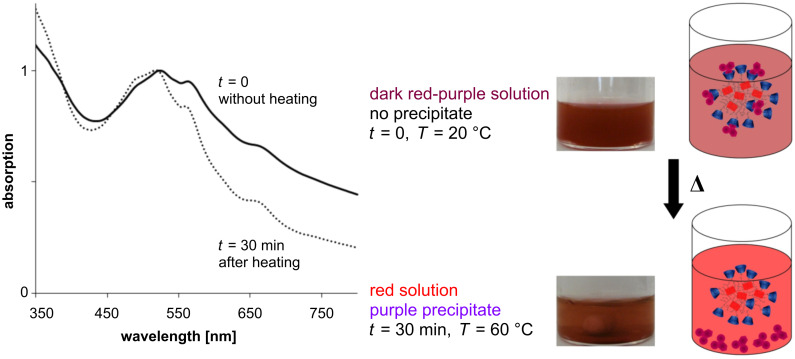
UV–vis spectra of complex **6** in solution (pH 6) before heating (solid line) and after 30 min heating at 60 °C (dotted line), and corresponding photos and schematic illustrations of the solution.

The absorption plot of complex **6** (a combination of the red and the purple dyes) exhibits maxima at 524, 564 and 656 nm. To show the separate release of dye **2**, complex **6** was heated for 30 min at 60 °C. At pH 1–8 the absorption undergoes a hypsochromic shift and a decrease of the maximum of the purple dye, resulting in a red solution (Figure 3).

**Figure 4:**
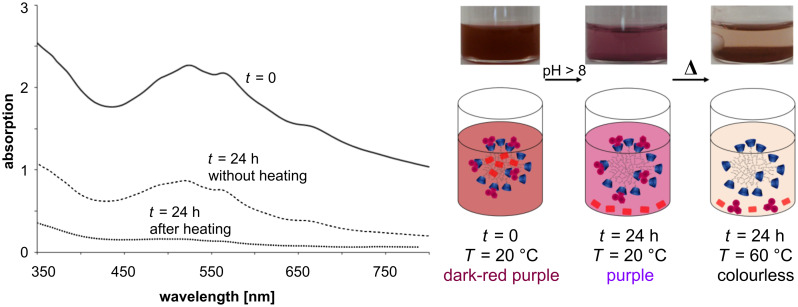
UV–vis spectra of complex **6** in basic solution (pH 10) before heating (solid line), after 24 h without heating (dashed line) and after 30 min of heating at 60 °C (dotted line), and corresponding photos and schematic illustrations of the solution.

In contrast, the separate release of dye **1** was forced by pH adjustment. At pH 9–14 the characteristic maximum of the red dye **1** (524 nm) decreases and a bathochromic shift is observed (Figure 4).

Furthermore, a complete release (~1 mg of each dye from 5 mg of complex **6**) of both dyes was achieved by heating the solution of complex **6** at pH 9–14 (Figure 4). Hence, two different components can be enclosed in one polymer and released separately or simultaneous in response to different external stimuli.

## Conclusion

The controlled release of different dyes from a drug delivery system based on hyperbranched polyethylenimine was investigated. The ionic interactions between the PEI scaffold and the hydroxy moieties of 5,8-dichloro-1,4-dihydroxyanthraquinone (AQ-OH) can be dissolved by pH adjustment. In contrast, the adamantyl modified compound 1,4-di-*N*-adamantylaminoanthraquinone (AQ-Ada) forms a host–guest complex with the PEI attached CDs and can be released by heating. These dyes serve as model compounds to illustrate the effectiveness of PEI-CD as drug delivery system in which two different components can be enclosed and released, either separately or simultaneously, in response to different external stimuli.

## Experimental

**Measurements:** Dynamic Light Scattering (DLS) experiments were carried out with a Malvern Nano ZS ZEN 3600 in a temperature range from 6 to 50 °C. The particle size distribution was derived by deconvoluting the measured intensity autocorrelation function of the sample with the NNLS general purpose mode algorithm included in the DTS software. UV–vis spectra were recorded in water using the UV 540 system of the company Unicam.

**Materials:** All reagents used were commercially available and were used without further purification.

**Synthesis of the host-molecule hyperbranched polyethylenimine covalently attached with β-cyclodextrin (3):** Polymer **3** was synthesized according to [16] (Supporting Information File 1, Scheme S1). Briefly, compound **3** can be obtained through polymer-analogous amidation with 5-hexynoic acid and subsequent reaction with mono-(6-azido-6-desoxy)-β-cyclodextrin by click reaction under microwave assisted conditions.

**Synthesis of 1,4-di-*N*-adamantylaminoanthraquinone (2): 2** was synthesized according to literature procedure [27] (Supporting Information File 1, Scheme S2). Briefly, 1,4-dichloroanthraquinone, adamantylamine and disodium hydrogen phosphate were suspended in *N*-methylpyrrolidone and heated for several hours. The reaction mixture was poured into water, filtered off and dried.

**Synthesis of host–guest complexes 4, 5 and 6: 4, 5:** In 20 mL water, 0.15 g of **3** and 0.05 g of the anthraquinone derivative (either **1** or **2**) were added and stirred for 24 h; **6:** In 20 mL water, 0.15 g of **3** and 0.05 g of both anthraquinone derivatives **1** and **2** were added and stirred for 24 h.

**Preparation for UV–vis spectroscopy:** 0.5 mL of the solutions **4, 5** or **6** was added to 3 mL of different aqueous solutions (of sodium hydroxide or hydrochloric acid) with pH values from 1 to 14.

## Supporting Information

Supporting Information features detailed data on syntheses, and DLS and UV–vis measurements.

File 1 Experimental details.
