# Supplementary material for: Hyperbranched polyethylenimine bearing cyclodextrin moieties showing temperature and pH controlled dye release
Source: Beilstein J Org Chem. 2011 Aug 18;7:1130–4. doi: 10.3762/bjoc.7.130 (PMC3167151; doi:10.3762/bjoc.7.130)
Supplement: File 1 — Experimental details. [file Beilstein_J_Org_Chem-07-1130-s001.pdf]

Supporting Information

for

Hyperbranched polyethylenimine bearing cyclodextrin moieties showing temperature and pH controlled dye release

Indra Böhm[‡], Susanne Katharina Kreth[‡], Helmut Ritter*[,‡]

Address: Institut für Organische und Makromolekulare Chemie II, Heinrich-Heine-Universität Düsseldorf, Universitätsstraße 1, D-40225 Düsseldorf, Germany Fax: (+49) 211 8115840

Email: Helmut Ritter* - h.ritter@uni-duesseldorf.de

*Corresponding author

[‡]Equal contributors

Experimental details

Synthesis of cyclodextrin modified hyperbranched PEI 3

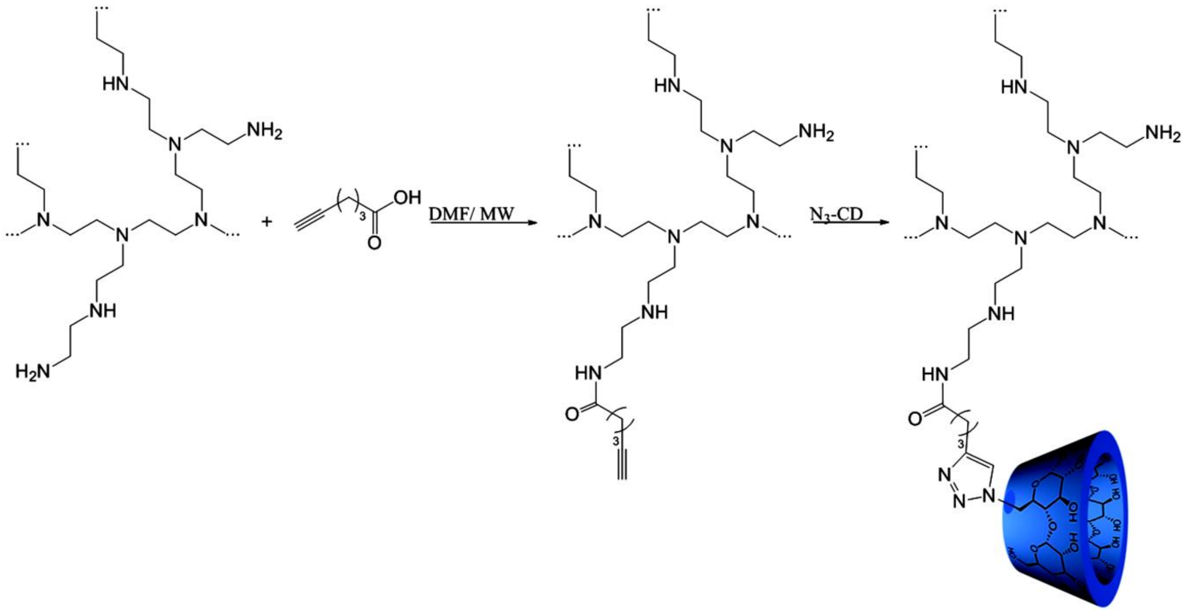

Scheme S1: Synthesis of **3**

Commercially available hyperbranched PEI (M_w = 10.000 g/mol) was employed. The polymer-analogous amidation with 5-hexynoic acid was implemented under microwave conditions. A solution of 5-hexynoic acid (1 g, 0.89 mmol) and PEI (1.12 g) in 8 mL DMF was added to a pressure-resistant test tube. The tube was sealed and placed in the CEM monomode microwave and irradiated at 85 °C and 60 W for 30 min. The subsequent reaction was implemented, without isolation of the intermediate, by adding sodium ascorbate (36 mg, 0.02 mmol), copper(II) sulfate pentahydrate (14 mg, 0.01 mmol) and mono-(6-azido-6-desoxy)-*β*-cyclodextrin (2.12 g, 0.18 mmol) to the solution. The tube was sealed and placed again in the CEM monomode microwave and irradiated at 85 °C and 60 W for 60 min. The product PEI-CD **1** was precipitated with acetone (100 mL) and dialyzed (MWCO 3500) to separate the polymer **1** from the catalysts and the monomers. The product was freeze-dried to afford 92% of pure product as a brownish powder.

3: UV (water): λ_{max} (ε) = 300 nm (1200 $L\cdot mol^{-1}\cdot cm^{-1}$); ^{1}H NMR (DMSO-$d_6$): δ = 083–0.84 (8H, -$CH_2$-), 0.93 (3H, C≡CH), 1.13–1.27 (12H, -CH_2-), 1.35–1.41 (4H, -CH_2-), 1.46 (8H,-CH_2-), 1.62–1.65 (4H,

-CH_2-), 1.78–1.81 (8H, -CH_2-), 1.35–1.41 (4H, -CH_2-), 2.05–2.08 (16H, -CH_2-), 2.10–2.17 (16H, -CH_2-), 2.22–2.25 (8H, -CH_2-), 2.33–2.35 (8H, -CH_2-), 2.78 (12H, -CH_2-), 2.80 (4H, -CH_2-), 2.93 (12H, -CH_2-), 3.34 (14H, H-2, 4), 3.56–3.63 (br, 28H, H-3, 5, 6), 4.49 (br, 6H, OH-6), 4.82 (d, 7H, H-1), 5.76 (14H, OH-2, 3), 7.79 (1H, -CH-), 8.02 (1H, -NH); IR: 3 304 (OH), 2 927 (CH), 2 825 (CH), 1 651 (Amid I), 1 546 (Amid II), 1 401 (OH), 1 151 (CN), 1 079 (OH), 1 028 (CH), 939, 853, 753 (NH), 703 (CH) cm^{-1}; Elemental analysis: C 50.32, H 7.84, N 10.93%.

Synthesis of dye 2

3.62 mmol of the 1,4-dichloroanthraquinone (synthesized according to literature procedure [1]), 1.71 g (11.3 mmol) adamantylamine and 1.18 g (8.31 mmol) disodium hydrogen phosphate were suspended in 4 mL *N*-methylpyrrolidone and heated to 180 °C for 12 h. The reaction mixture was poured into 100 mL of water and filtered off. Drying at 60 °C for 24 h gave the corresponding dye in a yield of 60 %.

2: UV/Vis (chloroform): λ_{max} (ε) = 524 nm; IR: 2 905, 2 848 $\nu(C\text{-}H)_{adamantyl}$, 1 706 ν(C=O), 1 598 $\nu(C{=}O)_{anthraquinone}$, 1 516 δ(N-H), 1 452 $\delta(C\text{-}H)_{asym}$, 1 306 $\delta(C\text{-}H)_{sym}$, 810 $\nu(C\text{-}H)_{aromatic}$ out-of-plane deformation vibrations (2 neighbouring H atoms), 721 $\nu(C\text{-}H)_{aromatic}$ out-of-plane deformation vibrations (4 neighbouring H atoms) cm^{-1}; ^{1}H-NMR ($CDCl_3$): δ = 8.14 (m, 2H, H1), 7.65 (m, 2H, H2), 7.31 (m, 2H, H3), 1.70 and 2.11 (m, 15H, adamantyl) ppm; MS (EI): m/z = 506 ($[M]^{+\bullet}$).

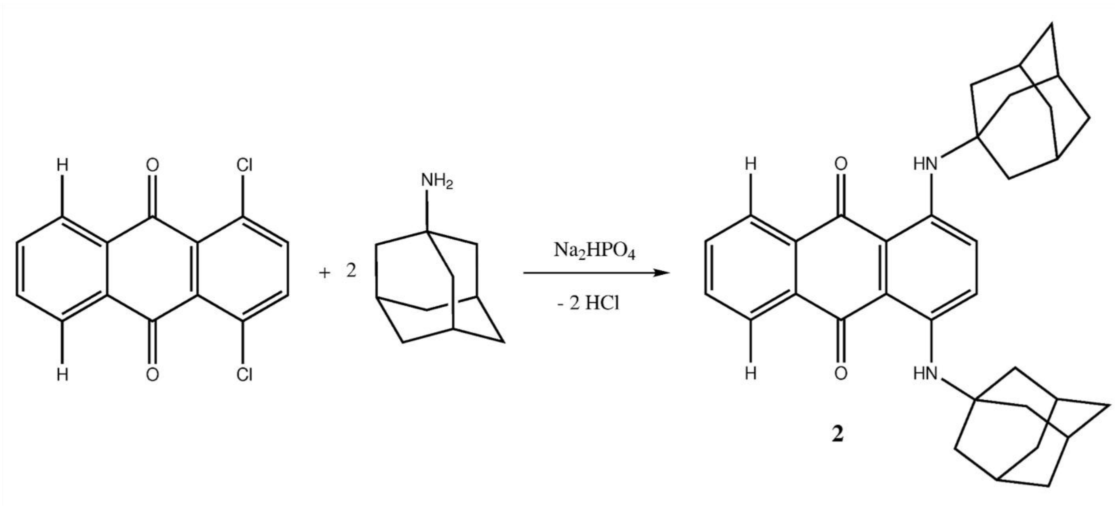

Scheme S2: Synthesis of adamantyl-containing dyes **2**

DLS of salt 4, PEI with 1, complex 5, compound 3 and RAMEB-CD with 2

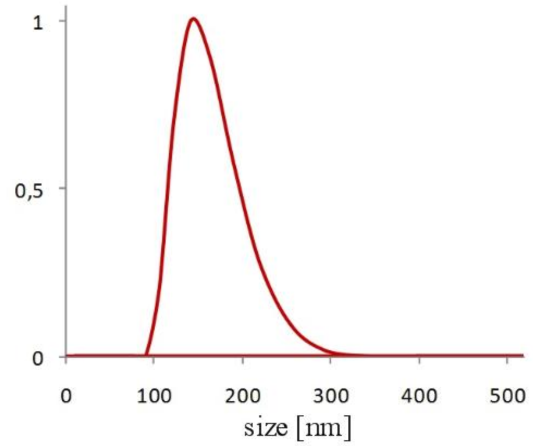

Figure S1: d_H of salt **4** (compound **3** with a d_H of 8 nm was combined with dye **1** leading to a d_H of 163 nm) (C_p= 0.5 g/L, in H_2O, pH 7, 25°C).

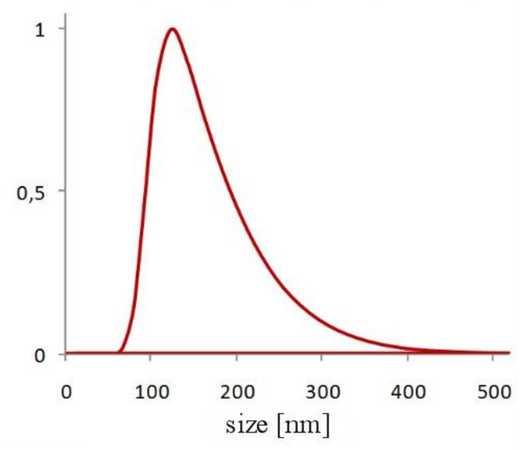

Figure S2: Unmodified PEI with a d_H of 4 nm was combined with dye **1** leading to a d_H of 152 nm (C_p= 0.5 g/L, in H_2O, pH 7, 25°C).

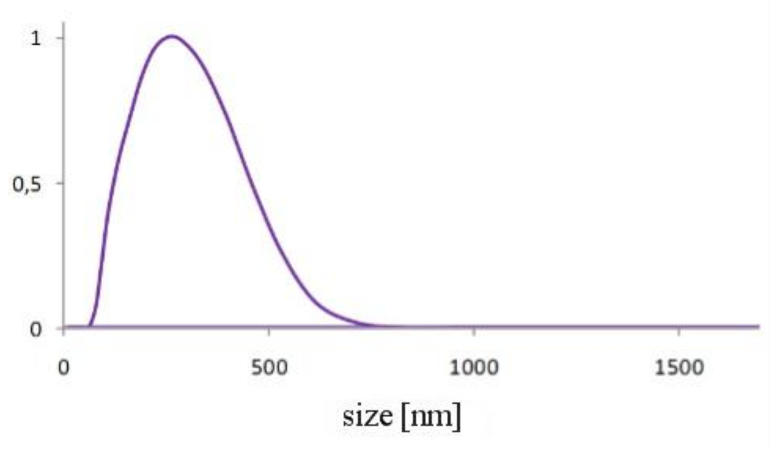

Figure S3: d_H of complex **5** (compound **3** with a d_H from 8 nm was combined with dye **2** leading to a d_H 260 nm) (C_p= 0.5 g/L, in H_2O, pH 7, 25°C).

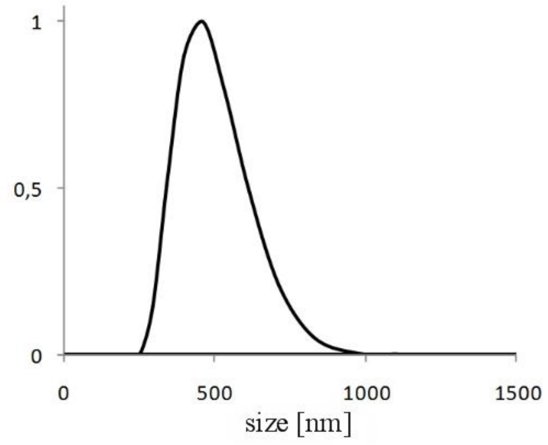

Figure S4: d_H of complex **6** (d_H of 480 nm which verifies an inclusion of both dyes (**1** and **2**)) (C_p= 0.5 g/L, in H_2O, pH 7, 25°C).

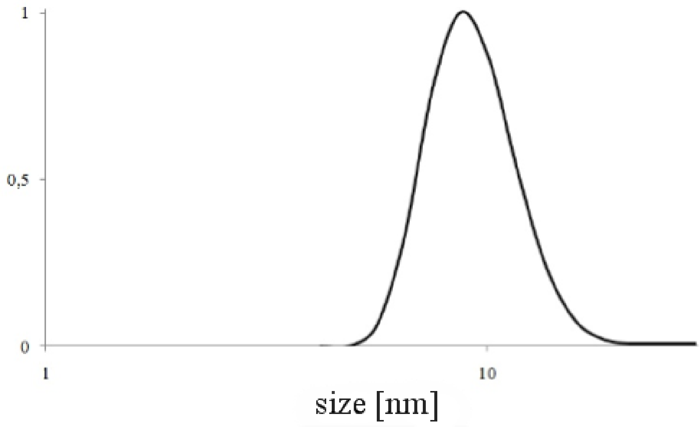

Figure S5: d_H of compound **3** (C_p= 0.5 g/L, in H_2O, pH 7, 25°C).

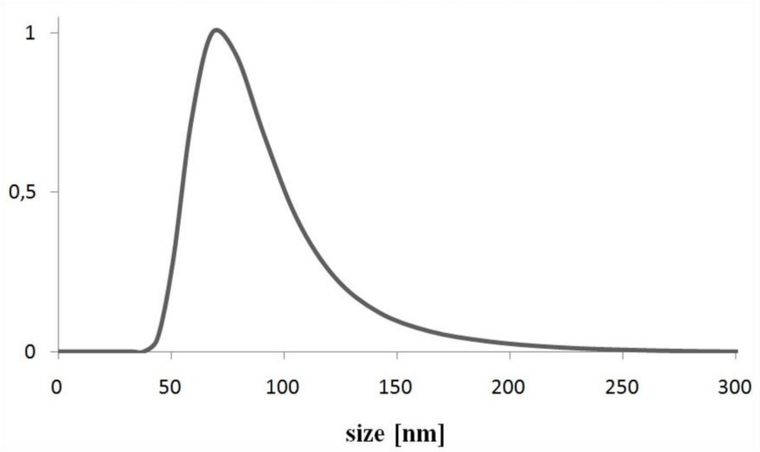

Figure S6: DLS measurements of RAMEB-CD and dye **2** showing a d_H of 68 nm (C_p= 0.5 g/L, in H_2O, pH 7, 25°C).

UV–vis spectra and photo of complex 5

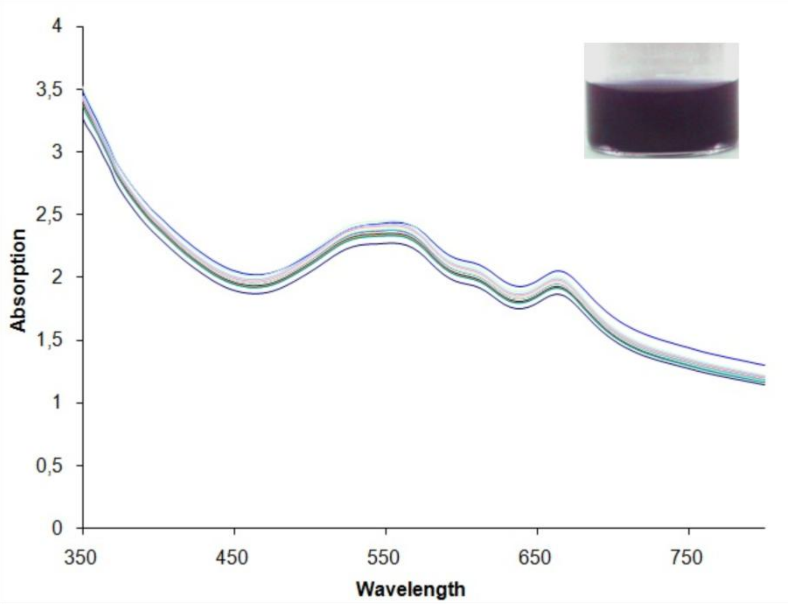

Figure S7: UV/Vis spectra and photo of complex **5** in solution (pH 1–14 at day 1) showing no sensitivity to pH change.

[1] Schönhagen, H.; Schmitz, R. Process for the preparation of 1,4-dichloroanthraquinone. DE3513981A1, October 30, 1986.
